# Social and knowledge diversity in forest education: Vital for the world’s forests

**DOI:** 10.1007/s13280-024-02104-6

**Published:** 2024-12-05

**Authors:** Mika Rekola, Andrew B. Taber, Terry L. Sharik, John A. Parrotta, Michael J. Dockry, Folaranmi D. Babalola, Tara L. Bal, David Ganz, Marta Gruca, Manuel R. Guariguata, James Kungu, Pipiet Larasatie, Anne Nevgi, Sandra Rodriguez-Piñeros, Sirichai Saengcharnchai, Niclas Sandström, Khalil Walji

**Affiliations:** 1 Department of Forest Sciences, University of Helsinki, P.O. Box 27, 00014 Helsinki, Finland; 2 Independent Researcher, Buenos Aires, Argentina; 3 College of Forest Resources and Environmental Science, Michigan Technological University, Houghton, MI 49931-1295 USA; 4 Independent Researcher, 7031 Haycock Road, Falls Church, VA 22043 USA; 5 Department of Forest Resources, University of Minnesota, 115 Green Hall, 1530 Cleveland Ave. N, St. Paul, MN 55108 USA; 6 Department of Forest Resources Management, Faculty of Agriculture, University of Ilorin, Ilorin, 240003 Kwara State Nigeria; 7 CFRES, Michigan Technological University, Houghton, MI 4993 USA; 8 RECOFTC, 50 Phahonyothin Rd, Lat Yao, Chatuchak, Bangkok, 10900 Thailand; 9 Food and Agriculture Organization of the United Nations, Viale delle Terme di Caracalla, 00153 Rome, RM Italy; 10 Center for International Forestry Research and World Agroforestry (CIFOR-ICRAF), Lima, Peru; 11 School of Environmental Studies, Planning and Management, Kenyatta University, P.O. Box 43844, Nairobi, Kenya; 12 University of Arkansas at Monticello, 346 University Drive, Monticello, AR USA; 13 Forum4edu, Faculty of Education, University of Helsinki, P.O. Box 9, 00014 Helsinki, Finland; 14 Universidad Autónoma de Chihuahua, Periferico Francisco R. Almada Km 1, C.P. 31453 Chihuahua, Chih Mexico; 15 Faculty of Education, University of Helsinki, P.O. Box 9, 00014 Helsinki, Finland; 16 Center for International Forestry Research and World Agroforestry (CIFOR-ICRAF), Nairobi, Kenya; 17 Villa de Leyva, Colombia

**Keywords:** Forest education, Indigenous knowledge, Knowledge diversity, Social diversity

## Abstract

A global assessment of the status of tertiary, vocational, and technical forest education and training found deficits in inclusion of knowledge and student diversity. Coverage of *forest services and cultural and social issues* was characterized as weak in the curricula of many programs. The inclusion of traditional and Indigenous knowledge was frequently poor or absent. Gaps were found in enrollment at tertiary education levels with respect to diversity by gender, race/ethnicity, and other societal groups. If unaddressed, forest researchers, professionals, and workers will continue to lack familiarity with different knowledge systems and the importance of inclusive representation. Improvements in forest education related curricula, research, monitoring, policy, recruitment, and promotion are recommended. Without remedial action to build a representative, skilled, and knowledgeable workforce, prospects for forests to meet local, national, and global goals are at risk. Improved social and knowledge diversity in forest education is paramount for the future of forests.

## Introduction

The world’s forests from the taiga to the tropics are extremely diverse in terms of both ecosystems and human management regimes. The sustainable management, restoration, and protection of these ecosystems are dependent on a diverse array of stakeholders, such as consumers of forest products, landowners, natural resource users and managers, conservators, policy makers, educators, and researchers. Moreover, the sustainable management of forests is contingent on alignment with the interests and values, and especially the contributions, of those who live in or near forests, especially local communities which are often Indigenous Peoples (Parrotta and Trosper 2012; Rodriguez-Piñeros et al. 2012; Garnett et al. 2018). Fundamentally, the sustainable development of the Global Forest estate relies on forest managers—forest professionals, communities including Indigenous Peoples, smallholders, and others—having diverse skills and knowledge, incorporating both Western science and Indigenous/traditional perspectives. Successful forest management also requires incorporation of gender, ethnic, racial, and cultural diversity in forest-related jobs and careers. Full coverage of under-represented and underserved groups of relevance to the forest sector is essential, as found for other sectors of human society (Page 2007).

A recent global assessment of forest education and curricula has revealed major deficits in the quality and coverage of university, college, technical, and vocational education and training around the world (Rekola and Sharik 2022; Wilenius et al. 2024). This is occurring despite increases in the overall number of graduates from university and technical forestry programs (FAO 2020). However, regional differences exist, and the data on graduate numbers and qualifications are more complex than they were previously as multi/interdisciplinary programs, such as environmental management have replaced traditional forestry programs (Sharik et al. 2015; Jegatheswaren et al. 2018). As a consequence, effective forest management and conservation to meet national and global environmental and societal goals are at risk from not having enough well educated and trained forest professionals, researchers, and workforces. While exceptional institutions exist in many regions, the assessment found two main challenges.

First, the assessment revealed a weakness in most education and training programs in their coverage of subject matter related to the thematic area of *forest services and cultural and social issues*, including the following topics: wood as renewable energy, forests-based recreation, traditional and/or Indigenous forest-related knowledge, cultural values of forests and trees, forests and human health, forests, trees and gender issues, and forests, trees, and ethnicity issues. Recognition and inclusion of traditional and Indigenous forest-related knowledge were particularly poor or absent as reported by students, recent graduates, teachers, and practicing professionals in most parts of the world. This is of particular concern given the cultural and practical importance of Indigenous forest-related knowledge to forest ecosystems, resources, and their management and use. Furthermore, challenges to pass this knowledge forward as well as document it is confounded by often weak protection of the intellectual property rights of Indigenous Peoples (ILO 2022). This deficit in *knowledge diversity* is resulting in forest professionals missing invaluable opportunities to learn from a variety of knowledge systems, and from the unique capacities in forest management and conservation of traditional and Indigenous cultures around the world.

Second, the assessment identified gaps in tertiary education enrollment with respect to diversity in gender, race/ethnicity, and other societal groups. Many current female and under-represented racial/ethnic students hesitate to enroll in forest education and training programs (Bal et al. 2020) and some of those that do may experience difficulties in finding decent employment in forest-related fields (Sharik et al. 2015). Furthermore, many lack opportunities to access formal tertiary forest education in the first place. *Social diversity* shortfalls in forest-related programs are resulting in sectoral under-representation and underservice in education, practice, and policy. Examples include under-recognition of forest economic activities that principally involve women, unsafe working environments for women, and lack of representation among state officials governing lands used by Indigenous Peoples.

These two deficits have consequences for the world's forests and trees and the billions of people who depend on their resources, goods, and services. A greater appreciation of local forest people’s contexts, contributions, and time-tested approaches can help enhance the utilization of a broader spectrum of evidence-based Western science and traditional forest-related knowledge by forest managers as well as expand and refine research agendas. Further, unless these deficits are addressed, education and training will continue to underserve those who live in and around forests and local people, especially Indigenous Peoples, who commonly lack agency in knowledge generation and decision-making about forests. Addressing these deficits can enhance the prospects that forests will thrive, continue to meet local, national, and global needs, and contribute to the fulfillment of the many United Nations Sustainable Development Goals related to forests.

## A global assessment

A global assessment of forest education was conducted in 2020, primarily through an online survey of students and recent graduates, forest professionals, teachers, and program administrators (Rekola and Sharik 2022; Wilenius et al. 2024). The number of responses was similar across these respondent groups, ranging from 840 student responses to 968 professional responses. The regional variation in response numbers was high, with the largest share of responses, 32%, coming from Latin America and the Caribbean (Table 1).

**Table 1:** Respondents by region and respondent groups in the Global Forest Education Assessment. *Source*: Rekola and Sharik (2022)

	Professionals	Teachers	Students	Total	Percentage of total (%)
1 Africa (AF)	136	117	129	382	14
2 Asia and the Pacific (AP)	180	145	113	438	16,2
3 Europe (EU)	170	173	118	461	17
4 Latin America and the Caribbean (LA)	274	333	289	896	32
5 Near East and North Africa (NENA)	34	36	5	75	2,8
6 North America (NA)	144	159	186	489	18
Total	968	963	840	2741	100

The survey covered primary and secondary schooling, universities and colleges, and technical and vocational education and training (TVET) in six regions: Africa (AF), Asia and Pacific (AP), Europe (EU), Latin America and the Caribbean (LAC), Near East and North Africa (NENA), and North America (NA). Of 2741 respondents, 38 percent were women and 11 percent self-identified as racial and/or ethnic minorities. However, it is likely that the digital survey did not reach various relevant groups living without ready access to modern communication facilities. In addition, expert consultations involved nearly 500 forest-related experts covering all regions of the world. Findings of the assessment were discussed at the International Conference on Forest Education held virtually in June 2021 (FAO 2021).

### Knowledge diversity

**Fig. 1:**
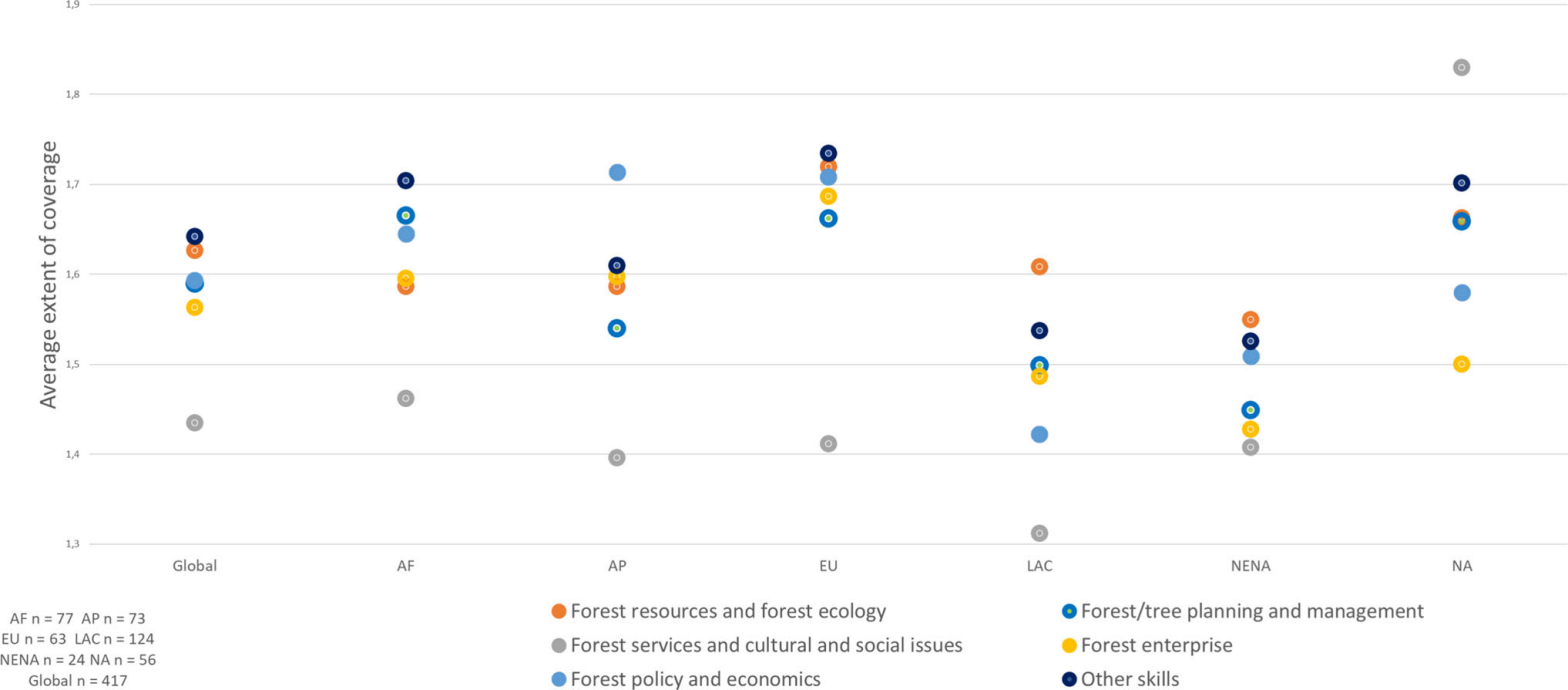
Coverage of forest-related themes in TVET education. Scale: 1 = inadequately covered, 2 = sufficiently covered, and 3 = excessively covered. *Source*: Rekola and Sharik (2022)

The global assessment examined coverage in forest education and training in six thematic knowledge areas (see Fig. 1). Each thematic area consisted of several topics; for instance, forest services and cultural and social issues had seven topics. The question in the survey was formulated as follows: “To what extent are the following topics and skills covered in the forest programme?” This question instrument was based on the one hand on so-called gap analysis (Lampley 2001; Arevalo 2010; Jackson 2011) and on the other hand on the curriculum coverage approach (Oketch et al 2012; Petersen et al 2020). Respondents rated the coverage of each topic using a five-point scale, i.e., “inadequately covered, sufficiently covered, excessively covered, unable to answer, and not applicable.” The main idea of the instrument was to identify the topics and skills that need more attention in education programs. The purpose was not to rank the importance of the topics and skills as such.

*TVET* results were based on 417 responses, primarily representing perceptions of professionals and teachers, with relatively few responses from students. Globally, findings indicated that several knowledge themes were insufficiently covered in TVET curricula, with six being poorly covered in Africa and the Asia and Pacific regions (Fig. 1). *Forest services and cultural and social issues* had the lowest coverage in all regions except North America, where respondents indicated that the coverage was high. Coverage of the traditional and Indigenous forest-related knowledge topic under this theme was scored as low across all regions.

**Fig. 2:**
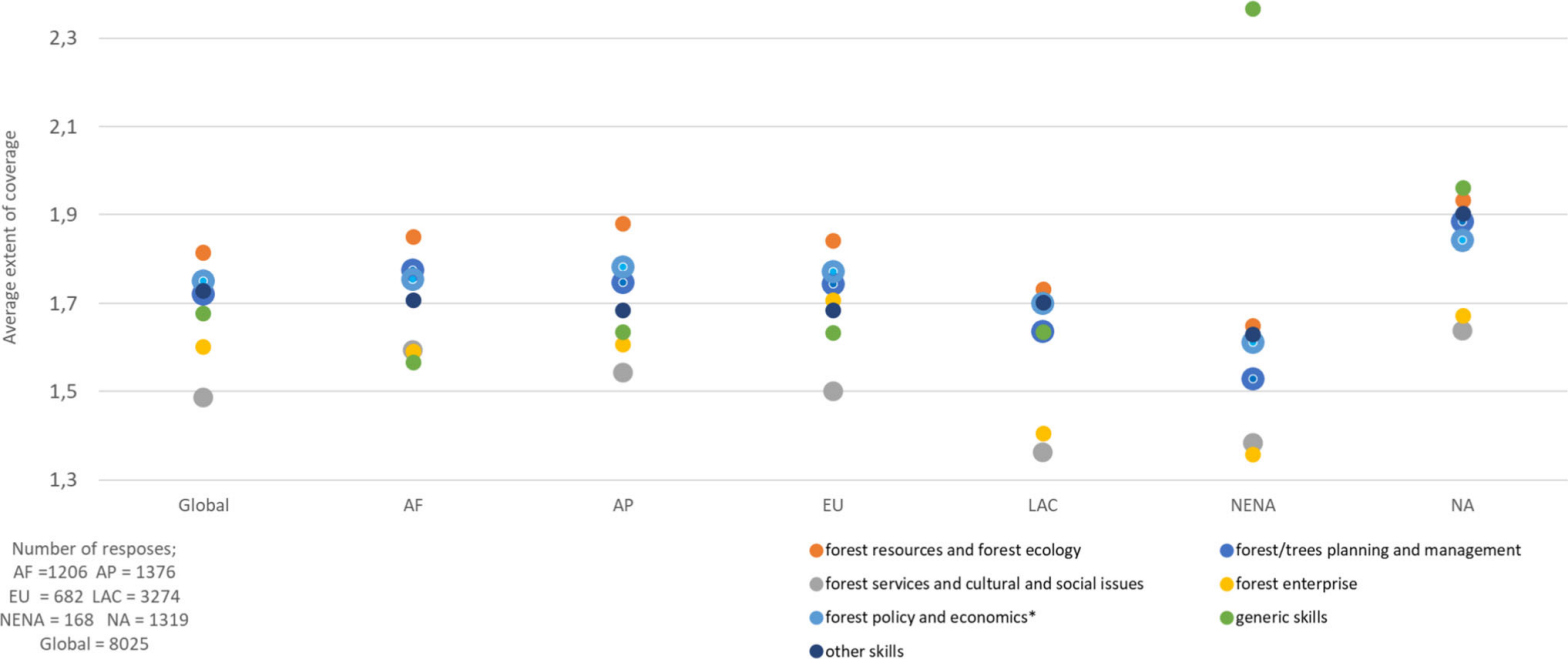
Coverage of forest-related themes in bachelor’s programs of universities and colleges. Scale: 1 = inadequately covered, 2 = sufficiently covered, and 3 = excessively covered. *Source*: Rekola and Sharik (2022)

*University and College* results at the bachelor's degree level, based on 1007 responses, showed that coverage of most of the themes was judged by respondents to be inadequately to sufficiently covered (Fig. 2). Again, the lowest scores were reported for the theme *forest services and cultural and social issues*, with coverage of Traditional and Indigenous forest-related knowledge; forests, trees, and gender issues; and forests, trees, and ethnicity issues being lower than average. Other topics under this theme were wood as renewable energy; forest-based recreation; cultural values of forests and trees; and forests and human health.

At the master's and doctoral levels, ratings of the adequacy of coverage varied greatly among regions and respondent groups. Again, the theme that was considered least well covered globally was *forest services and cultural and social issues*. Within this theme, coverage was considered inadequate by respondents from all regions for three topics relevant to social and knowledge diversity, i.e., forests, trees, and gender; traditional and Indigenous forest-related knowledge; and cultural values of forests and trees.

The recognition, adoption, adaptation, and also generation of new forest-related knowledge by local people about forests and their management is of great importance, particularly in light of the rapidly changing climate and ecological and socioeconomic circumstances in forests around the world. Indigenous communities rely on this knowledge in the management of forests under their stewardship, which generations of experience has shown to be remarkably effective for maintaining healthy or intact ecosystems, high biodiversity levels, low deforestation rates, and effective ecological restoration (Adam and Kneeshaw 2008; Parrotta and Trosper 2012; Fa et al. 2020).

While this knowledge is often attached to specific geographical and cultural contexts in a certain time period—what might be appropriate in one place may not be applicable elsewhere—it, nevertheless, can prove invaluable to draw from for broader application.

Despite its increasingly recognized importance, the inclusion of traditional and Indigenous forest-related knowledge in education and training faces many challenges (Brondizio et al. 2021). A significant issue is how best to integrate, or impart in parallel, knowledge coming from different traditions such as from Indigenous Peoples (Lake et al. 2018). A further challenge is that in many local communities globally, intergenerational transmission is impeded for various reasons. Some Indigenous groups may not be willing to share their knowledge with others, particularly where Indigenous rights related to land and its management are compromised. The retention and sharing of traditional knowledge are further hampered in countries where intellectual property right regimes are inadequate and Free, Prior, and Informed Consent principles are not applied to ensure the interests of knowledge holders are protected (ILO 2022). Elders in many forest communities are often the only remaining holders of traditional knowledge related to forest ecosystems and their management, increasing the urgency of consensual recording of this knowledge and dissemination through education and training, especially education for Indigenous Peoples themselves.

### Social diversity

#### The gender dimension

Forest economies are diverse yet are often perceived as male-dominated due to higher profile production and marketing of high-value wood products (Lippe et al. 2022). Yet in many regions, women play major roles in forest management as well as in the collection, processing, and marketing of forest products for food security, nutrition, health, tourism, and social services. More than 1 billion people rely on forest resources for their livelihoods, especially Indigenous women and children (ILO 2019). The collection of fuel wood for cooking and water purification in developing countries has been mainly undertaken by women (Dovie et al. 2004). Women often hold unique knowledge and their preferences for diverse forest goods and services contribute to biodiversity conservation and can counteract habitat conversion pressures. They often advocate for equitable and sustainable solutions to forest-related environmental challenges. Their engagement in research, monitoring, and socio-economic studies has been shown to deliver more accurate and comprehensive results.

Women face challenges for enabling their roles in forests (FAO 2020; FAO 2022), which are amenable to improvement through forest education and training (Asher 2018; FAO 2020; Ota et al. 2024; Bitzer 2024). Gender balance is a persistent challenge for forest education, with women under-represented both in current forestry workforces and among students (Arevalo et al. 2012; Sharik et al. 2015; Bal and Sharik 2019a, b; Grubbström and Powell 2020). It has been reported that women are more hesitant to enroll in programs than men. However, increasing numbers of women taking part in forest education show that hesitancies are decreasing in some regions (FAO 2020; Bal et al. 2020, 2023; Burmann et al. 2022).

Despite positive trends, while the number and proportion of women in forest education have increased, 5–30% of respondents to the global assessment, primarily students, reported gender to be a factor in finding jobs upon graduation. In the Asia and Pacific Region, for instance, female graduates indicated that gender was “moderately” to “very much” a factor in their ability to obtain forest-related employment. In Europe, many teachers reported that gender strongly affected graduate employment prospects (Rekola and Sharik 2022).

Nevertheless, women are active in the forest sector with potential to surpass that of men in value, but their contributions are often unrecognized, and with opportunities being lost as a consequence (FAO 2018). The forest sector would clearly benefit from greater inclusion of women, along with greater recognition of their contributions, in forest education and training.

#### The case of indigenous peoples

Indigenous Peoples, who influence an estimated 28% of the world’s land area and more than 40 percent of the global protected area (Garnett et al 2018), represent distinct social and cultural groups who play a vital role in protecting, restoring, and sustainably using forests. Indigenous communities rely on their traditional knowledge in the management of forests under their stewardship, which generations of experience has shown to be remarkably effective for maintaining healthy or intact ecosystems, high biodiversity levels, low deforestation rates, and effective ecological restoration (Adam and Kneeshaw 2008; Parrotta and Trosper 2012; Fa et al. 2020).

Indigenous Peoples comprise almost 19% of the extreme poor but only six percent of the global population. Opportunities for them to access quality employment are often scarce, and they are 20% more likely to work in the informal economy than other workers. Globally, 47% of employed Indigenous adults have no formal education compared to 17% of other adults (ILO 2019). In many countries, their access to formal education, including forestry, is limited. The substantial contributions of Indigenous educational systems and knowledge transmission in Indigenous societies (e.g., elders to youth, village level, etc.) for forest management are generally poorly documented, appreciated, and supported.

Indigenous Peoples often lack agency in the development of forest education and training curricula and programs that meet their requirements and cultural values. Limitations on use of local languages and other bio-cultural contexts in formal education in many countries can impede sharing of traditional forest-related knowledge. Furthermore, forest-related education in many countries does not ensure that others working in forests are fully aware and appreciative of the needs, values, rights, and cultures of Indigenous Peoples (Rekola and Sharik 2022).

The global assessment online survey did not specifically address the forest education needs of Indigenous Peoples. Such an exercise would require their full leadership and engagement. Nevertheless, it is clear that forest-related education and training can contribute to insuring Indigenous Peoples have the tools to access formal education, build enterprises, obtain jobs, support livelihoods, manage forests, and sustain cultures. A specific session at the International Conference on Forest Education held in June 2021, in which Indigenous Peoples participated, generated a list of action items around traditional knowledge and forest education (Table 2).

**Table 2:** Action items concerning traditional knowledge and forest education generated by participants including Indigenous Peoples at the International Conference on Forest Education held in June 2021

*Forest education and traditional knowledge*
Incorporation of traditional knowledge into forestry curricula and training activities must be founded on genuine mutual respect for differences between traditional and scientific knowledge systems and adhere to ethical research and knowledge dissemination standards Efforts must be made to engage traditional knowledge holders and institutions in development of forest curricula and build solid trust-based partnerships following participatory processes Fe and allowing time and space for joint planning, testing, reflecting, and implementing Supportive policies need to be in place to allow development of local curricula incorporating inputs from various knowledge holders, including traditional holders, and development of inclusive educational tools Forest education systems must pay due attention to safeguard consensual data collection, documentation, and sharing to protect intellectual property and sacred traditional knowledge Forest education systems should raise awareness on the importance of protecting intellectual property rights and empower communities to promote their traditional knowledge, control its uses and access to benefits, and exercise their right to Free, Prior, and Informed Consent
*Traditional knowledge systems and its management*
Traditional knowledge needs to be recognized as a distinct system of knowledge that requires handling and categorization that are different from those applied by the scientific system of knowledge management Efforts must be made to stimulate and create interest among youth in their cultural heritage, linguistic diversity, and facilitate intergenerational knowledge transfer while reaffirming the status of elders as knowledge holders Gender differences among traditional knowledge holders should be recognized and special attention paid to engage with both Indigenous men and women experts Global effort is urgently needed to identify, document, preserve, and protect traditional knowledge. Innovative approaches are required to facilitate access to knowledge for educational purposes while creating a level of transparency that can serve to minimize the risk of exploitation Concrete actions should be identified to capitalize on existing frameworks such as Open Science and the upcoming International Decade of Indigenous Languages (2022–2032) Environmental stewardship of Indigenous Peoples and local communities must be recognized and affirmed, and policies and programs should focus on preserving and protecting traditional knowledge and associated cultural and biological diversity

The listed items are rearranged here based on FAO, IUFRO, and ITTO (2021)

#### Other under-represented and underserved groups

All regions have under-represented and underserved groups or identities in populations of relevance to forests. The global assessment was confined to race/ethnicity, although other groupings are mentioned in the literature or were brought up in the consultations. These groups may be related to cultural or linguistic background, age, nationality, gender identity, sexual preference, religious preference, physical or emotional disabilities, urban versus rural residency, level of poverty, and military service, among others (US Department of Health and Human Services 2022). Examples in Latin America include Indigenous Peoples, peasant farmers, and others who have moved from forest to urban contexts due to poverty and violence (Álvarez 2003). Moreover, to our knowledge, there has been minimal examination to date of people with disabilities or lesbian, gay, bisexual, transgender, queer, and other gender, and sexual orientation identities (LGBTQ+) issues pertaining to forest education and training, or indeed forest practices and uses.

Around 40% of student respondents in Latin America, the Caribbean, and North America, and 15% of respondents in Asia and the Pacific, reported race and ethnicity as “moderately” to “very much” a factor in the access of graduates to forest-related employment (Rekola and Sharik 2022).

Data on these issues are limited from the assessment and in the literature. Rules and regulations for collecting statistics on racial/ethnic groups can be complex; for instance, in Europe, where collection of such data is prohibited in some countries—in part due to historical events. However, research in the US found that African and Asian descendants, Latinos, and Indigenous Peoples (i.e., Native Americans) were under-represented in forest-related academic programs and in the workforce compared to their numbers in the general population (Sharik et al. 2015). Other regions include various other under-represented racial/ethnic groups such as Sámi in northern Europe, and colonos and Afro-descendants in Latin America (Álvarez 2003).

Under-representation of certain racial/ethnic groups should not be surprising in some countries and regions where they have been discriminated against with respect to access to land and resources. Under such conditions, they may see little future in being part of the forest sector that is closely aligned with land and resources from which, in some cases, they have been alienated (Schelhas 2002; Sharik et al 2015).

## Conclusions: Actions for diversifying forest education

Based on the global assessment's findings, we have identified critical needs for improving inclusion in both knowledge and social diversity in forest education and training. If unaddressed, forest professionals and the forest sector workforce will continue to lack key knowledge and societal representation for meeting local, national, and global goals for forests. Research capacities and scope will also be limited. Solutions will be constrained for managing forest ecosystem services, including freshwater and carbon storage; conserving biodiversity; restoring ecosystems; securing forest contributions to food, nutrition, and health; sustaining production of wood and other forest goods; and maintaining societal and cultural services.

Below we identify four groups of recommended actions and associated roles for key actors in forest education and training to improve its diversity with respect to knowledge and social demographics.

### Targeted research and monitoring

The global assessment, the most thorough survey undertaken to date on the status of forest education and training, revealed information gaps that could be addressed through the development and implementation of a targeted research agenda. Many research questions related to social and knowledge diversity would require transdisciplinary research and monitoring. Priority topics include:

- *Documentation of traditional forest-related knowledge for inclusion in forest curricula.* This will require leadership by Indigenous and other forest-based peoples, especially given concerns for Intellectual Property Rights, the need for Free, Prior, and Informed Consent, and compliance with other standards. Western scientists, educators, trainers, donors, and others can support such communities in the ethical and responsible collection and use of such knowledge.
- *Typology and participation of different sectors of societies in forest education and training along with the consequences of under-representation*. Such information is vital to inform program design and curricula, understand constraints on participation, and devise recruitment strategies.
- *Longitudinal monitoring***.** More and finer-grained reporting and monitoring of forest education and training is needed by countries and international organizations. We encourage the United Nations Food and Agriculture Organization via the Global Forest Resources Assessment,^1^ and other initiatives such as the World Resources Institute's Global Forest Watch, to develop science-based criteria and methodologies, and implement tracking of progress towards improved knowledge and social diversity in forest education at national levels.

We note the need for extreme sensitivity when it comes to research and monitoring related to race/ethnicity and gender given ethical, political, and legal concerns in some countries and regions (Madden et al. 2016).

### Improvement in student recruitment and curricula

Updated national educational strategies, student recruitment approaches, and improved forest education curricula are needed to better serve under-represented and under-served groups, and enhance the role of traditional and Indigenous knowledge and other non-Western science-based knowledge systems in curricula. Academic organizations, networks of university and technical programs, forest education experts, and communities of practice all have key roles to play in this. The representation from all forest-relevant subsectors of society, as well as the effective engagement of Indigenous Peoples and recognition of their environmental stewardship and knowledge, will be crucial in these efforts. International bodies such as United Nations Forum on Forests, Collaborative Partnership on Forests and its Global Forest Education Initiative and the United Nations Educational, Scientific and Cultural Organization could offer guidance and standards.

### Improvements in delivery mechanisms

To ensure that forest education and training reach underserved sectors in forest education and training, we urge investment and prioritization in TVET-related approaches such as in situ forest extension and field school approaches which are easily accessible and adapted to local circumstances in terms of syllabi and languages used. Notably, Indigenous knowledge is language dependent, and many local languages are under threat today. The forest education community has an important role to play in supporting national education actors and agencies to improve forest-relevant curricula at primary and secondary school levels, and to increase awareness and interest in young people in forests and forest-related careers (Rodríguez-Piñeros et al. 2020).

### Raising awareness and changing attitudes

Addressing the deficits in knowledge and social inclusion in forest education will require significant and synergistic efforts across the forest sector, involving and reaching prospective students, current students (e.g., the International Forestry Student Association), program graduates, educators and trainers, program administrators, employers (public and private sector), NGOs (including those representing Indigenous Peoples), and the public. It will also require engagement with other educational and environmental agendas, including those under the Sustainable Development Goals, e.g., the UNESCO-led Education for Sustainable Development for 2030 agenda (Kanowski et al 2019).

The future of forests depends on young people of diverse backgrounds being drawn to forest-related education and training programs at all levels, and in turn to forest-related careers. Strategies to improve forest education in global or regional scales already exist, for instance, Collaborative Partnership on Forests (CPF), under the aegis of FAO, has launched a forest education initiative and the FOREST EUROPE process is running the Green Forest Jobs project. The number of success stories in grass-root level is not yet high, but a few do exist, and they are encouraging. Project Learning Tree in Canada, for instance, has placed over 7600 young adults in green jobs, including those in Indigenous communities and others facing additional barriers to employment (Project Learning Tree Canada 2024).

In this treatment, we have outlined actions needed to build a more representative, skilled, and knowledgeable workforce. In our view, the future of the world’s forests depends on a renaissance in forest education that ensures coverage of all relevant knowledge—whatever the source—and representation from all segments of society, including Indigenous Peoples and other marginalized groups. If deficits in contemporary forest education programs around the world, as described above, remain unaddressed, many forest researchers, professionals, workers, and entrepreneurs will continue to lack familiarity with different knowledge systems as well as the need for and importance of inclusive representation. This risks negative consequences for forests and agroforestry systems, forest and tree-related business, policy effectiveness, and societies, economies, and environmental sustainability more generally. Building a more inclusive forest education will better enable forests to meet local, national, and global goals now and in the future.

## Footnotes

1 https://www.fao.org/forest-resources-assessment/en/.
